# Lethal effects of ivermectin structures on malaria vectors and in silico analysis of interactions with their glutamate-gated chloride ion channels

**DOI:** 10.1038/s41598-026-39698-8

**Published:** 2026-02-10

**Authors:** Minh N. Nguyen, Andrew K. Jones, David Hotwagner, Pattarapon Khemrattrakool, Thitipong Hongsuwong, Borimas Hanboonkunupakarn, Podjanee Jittamala, Patchara Sriwichai, Joel Tarning, Kevin C. Kobylinski

**Affiliations:** 1 BioInformatics Institute (BII), Agency for Science, Technology and Research (A*STAR), 30 Biopolis Street, Singapore, 138671 Singapore; 2 Department of Biological and Medical Sciences, Oxford Brookes University, Oxford, OX3 0BP UK; 3 Mahidol Oxford Tropical Medicine Research Unit, Faculty of Tropical Medicine, Mahidol University, 420/6 Rajvithi Road, Ratchathewi, Bangkok, 10400 Thailand; 4 Department of Clinical Tropical Medicine, Faculty of Tropical Medicine, Mahidol University, 420/6 Rajvithi Road, Ratchathewi, Bangkok, 10400 Thailand; 5 Department of Tropical Hygiene, Faculty of Tropical Medicine, Mahidol University, 420/6 Rajvithi Road, Ratchathewi, Bangkok, 10400 Thailand; 6 Department of Medical Entomology, Faculty of Tropical Medicine, Mahidol University, 420/6 Rajvithi Road, Ratchathewi, Bangkok, 10400 Thailand; 7 Centre for Tropical Medicine and Global Health, Nuffield Department of Clinical Medicine, University of Oxford, Old Road Campus, Oxford, OX3 7BN UK; 8 Singapore, Singapore

**Keywords:** Ivermectin, *Anopheles*, Glutamate-gated chloride ion channel, Docking model, Biochemistry, Computational biology and bioinformatics, Drug discovery, Microbiology

## Abstract

Ivermectin is lethal to *Anopheles* mosquitoes making it a possible malaria control intervention. The primary mode of action of ivermectin occurs when it binds to the glutamate-gated chloride channel (GluCl), allowing for continuous flow of chloride leading to flaccid paralysis and death of the mosquito. In *Caenorhabditis elegans,* ivermectin is thought to open the GluCl channel when the M2-M3 loop forms Van der Waals bonds with the first sugar ring and aglycone structure of ivermectin. Here we investigate in *Anopheles dirus* and *Anopheles minimus* the mosquito-lethal effect of ivermectin (both sugar rings), monosaccharide (one sugar ring), and aglycone (no sugar rings) demonstrating full, partial, and no effect, respectively. The *Anopheles* GluCl protein sequences were determined and used to a create 3-D structural docking models. The docking models identified new binding interactions with a hydrogen bond forming between the second sugar ring hydroxyl group (4″-OH) and THR304 of the *Anopheles* GluCl M2-M3 loop. This hydrogen bond is possible due to a single substitution in the M2-M3 loop from *C. elegans* ILE273 to *Anopheles* THR304. The work presented here improves our understanding of *Anopheles* GluCl-ivermectin interactions as well as how ivermectin resistance could arise in the future.

**Supplementary Information:**

The online version contains supplementary material available at 10.1038/s41598-026-39698-8.

## Introduction

Ivermectin mass drug administration (MDA) is a possible new tool for malaria control and elimination as ivermectin-treated humans or livestock are lethal to blood-feeding *Anopheles*^1^. The proposed mode of action is that ivermectin binds at subunit interfaces of the glutamate-gated chloride (GluCl) ion channel in the lipid membrane bilayer, which distorts the channel from a closed to an open state^2^, allowing chloride to continuously flow through the channel hyperpolarizing the cell which leads to the paralysis of the nematode or arthropod musculature and possibly death^3–6^. The GluCl channel is a member of the Cys-loop ligand-gated ion channel superfamily, composed of five identical subunits, which are comprised of four transmembrane helixes (M1-M4)^7,8^. Protein crystallography of the free-living nematode *Caenorhabditis elegans* GluClα subunit demonstrates that the cyclohexene portion of ivermectin (C-7) binds to the (-) subunit M1 helix and C-5 to the ( +) subunit M2 helix while the spiroketal portion (0–14) binds to the ( +) subunit M3 helix, but more importantly the first saccharide sugar ring C-33 and C-32, and the lactone C-35 form Van der Waals (VDW) bonds with the GluCl extracellular M2-M3 loop (Supplemental Figure S1A). Ivermectin binding to the GluCl M2-M3 loop shifts the conformation of the M2 helix, forcing the GluCl channel to open^2^. In the fruit fly, *Drosophila melanogaster*, a single amino acid substitution in the GluCl M2-M3 loop from PRO299 to SER299 caused a 3.3-fold decrease in ivermectin lethal effect indicating that the M2-M3 loop is important for ivermectin activity in Diptera as well^5^.

Much of our knowledge of ivermectin-GluCl interactions is based on *C. elegans* and *D. melanogaster*, which have inherent differences from *Anopheles* mosquitoes. In *C. elegans* there are several GluCl subunit-encoding genes^9^, two of which are CeGluClα and CeGluClβ, with the CeGluClα sensitive to ivermectin but not glutamate, while CeGluClβ is sensitive to glutamate but not ivermectin^3^. Ivermectin is a partial allosteric agonist of CeGluClα, once the channel is opened by ivermectin then glutamate is able to bind in the extracellular domain increasing chloride flow through the channel by 30–70%^3^. However, in *D. melanogaster* there is only one subunit, DmGluClα, that is sensitive to both glutamate and ivermectin. DmGluClα channel conformation is irreversibly altered by ivermectin, therefore ivermectin is an allosteric agonist of DmGluClα^4,5^. The primary African malaria vector, *Anopheles gambiae*, also has one GluCl subunit (AgGluCl), which is sensitive to both glutamate and ivermectin. However, ivermectin is only a partial allosteric agonist of AgGluCl^10^. AgGluCl shares 47% protein sequence identity with CeGluClα and 93% with DmGluClα which indicates that further investigation into *Anopheles* GluCl-ivermectin interaction is warranted.

The primary Southeast Asian malaria vectors, *Anopheles dirus* and *Anopheles minimus*, are both sensitive to ivermectin. While *An. dirus* is one of the most ivermectin-tolerant species, *An. minimus* is one of the most ivermectin-sensitive species across the globe^1^, differing in susceptibility by 6.9-fold^11^, making them ideal species to evaluate in tandem. The three most abundant metabolites of ivermectin found in humans (*i.e*. 3″-*O*-demethyl ivermectin, 4-hydroxymethyl ivermectin, and 3″-*O*-demethyl, 4-hydroxymethyl ivermectin) had equivalent mosquito-lethal effect as parent ivermectin in *An. dirus*, *An. minimus*^11^, and *Anopheles stephensi*^12^. Here we assess the mosquito-lethal effect of various ivermectin structures (*i.e.* ivermectin, monosaccharide, aglycone) on *An. dirus* and *An. minimus*, and develop in silico ivermectin docking models to predict how these ivermectin structures and metabolites interact with *Anopheles* GluCl.

## Results

### Mosquito mortality results

**Fig. 1:**
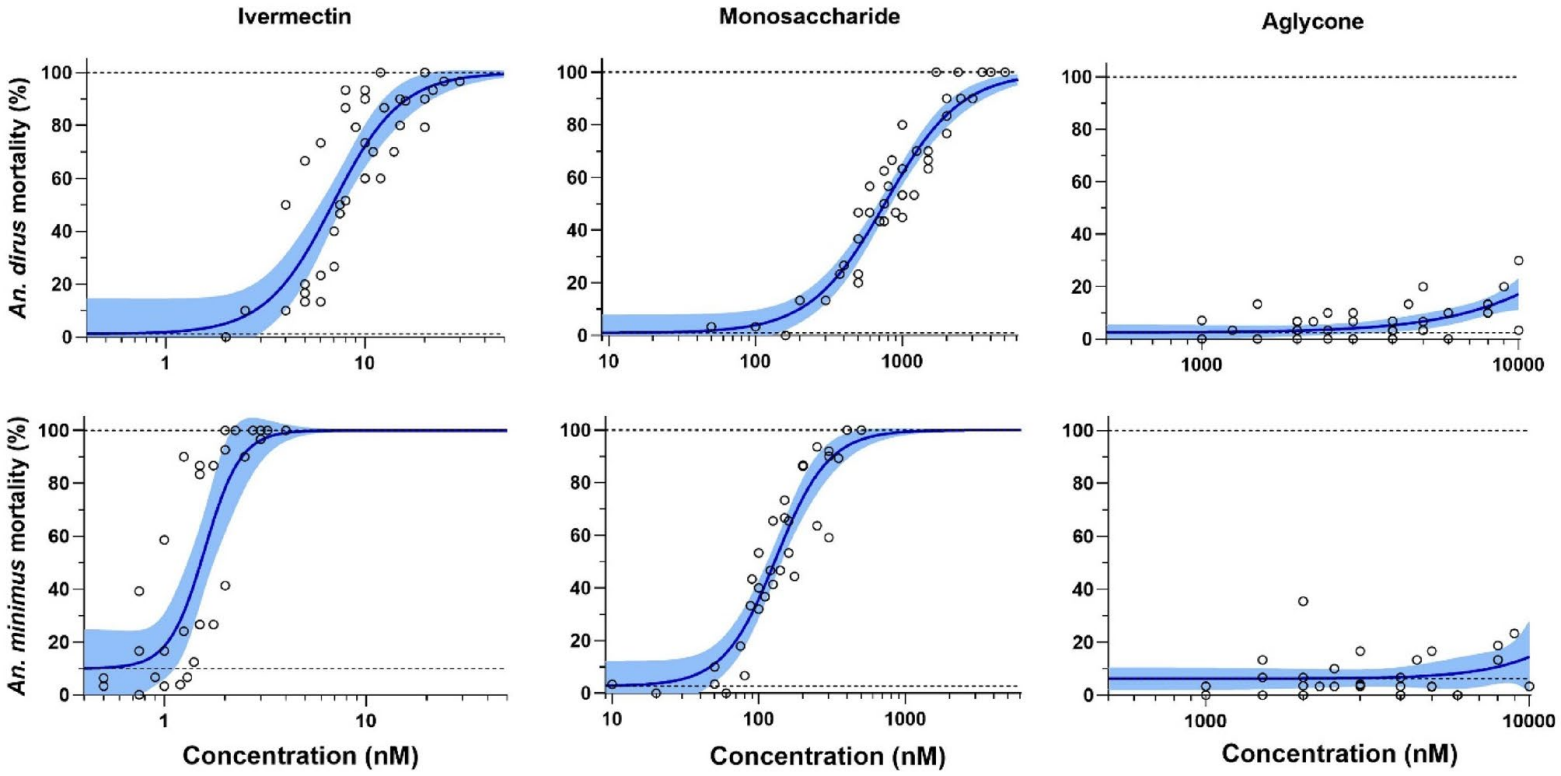
*Anopheles dirus* (top row) and *An. minimus* (bottom row) mortality results when fed ivermectin (left panels), monosaccharide (middle panels) and aglycone (right panels) in human blood. Open circles represent cumulative mosquito mortality at 10 days after blood meal ingestion. Solid lines represent the mean concentration–response relationship and the shaded area represents the 95% confidence interval associated with the nonlinear fit. Dashed black lines represent the fixed maximum effect of 100% mortality and the estimated minimum effect associated with baseline mortality observed from control mosquitoes.

A total of 4,043 *An. dirus* mosquitoes across five replicates were used to calculate the LC_50_ for various compounds including: ivermectin (n = 1,346), monosaccharide (n = 1,348), and aglycone (n = 1,349). A total of 3,114 *An. minimus* mosquitoes across four replicates were used to calculate the LC_50_ for various compounds including: ivermectin (n = 1,042), monosaccharide (n = 1,030), and aglycone (n = 1,042). Ivermectin was more lethal to *An. minimus* than to *An. dirus* by a factor of 4.4-fold (Fig. 1, Table 1). Monosaccharide was lethal to *An. dirus* and *An. minimus* but there was a 111-fold and 83-fold difference in susceptibility compared to ivermectin, respectively (Fig. 1, Table 1). For aglycone, LC_50_ values for *An. dirus* and *An. minimus* were estimated to be beyond the observed data, so this estimate may not be biologically relevant and for *An. minimus* the model could not predict the 95% confidence interval (CI) (Fig. 1, Table 1).

**Table 1:** *An. dirus* and *An. minimus* LC_50_ values for ivermectin, monosaccharide, and aglycone calculated based on 10-day observation.

Species	Compound	LC_50_ (nM)	95% CI
*An. dirus*	Ivermectin	6.9	[5.7—8.2]
	Monosaccharide	766.9	[668.0—874.3]
	Aglycone	24,228.0	[13,242.0—166,034.0]
*An. minimus*	Ivermectin	1.6	[1.3—1.9]
	Monosaccharide	131.3	[114.8—150.6]
	Aglycone	22,657.0	[NE]

LC_50_ represents the lethal concentration that kills 50% of either *An. dirus* or *An. minimus* at ten days after a blood meal for each compound. Note, the Aglycone LC_50_ values were extrapolated as the mortality results never cross the 50% threshold and thus may not be accurate. NE, not possible to estimate.

### Amplification and sequencing of *An. dirus* and *An. minimus* GluCl

**Fig. 2:**
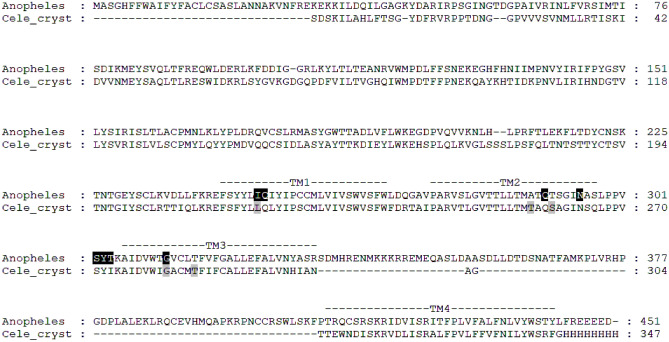
Protein sequence alignment of full length AnGluCl (*An. dirus*, *An. gambiae* or *An. minimus*) with truncated *C. elegans* CeGluClα used to generate crystal structure (Cele_cryst; Accession No. 4TNW_T). The four transmembrane domains (M1-M4), as previously denoted, are indicated with dashes. Residues implicated in ivermectin binding are highlighted in black shading and white text for AnGluCl, and grey shading for *C. elegans* GluCl^2^.

The cDNA open reading frames of *An. dirus* and *An. minimus* GluCl were amplified by reverse-transcriptase PCR. Analysis of the resulting amino acid sequences containing the alternatively spliced exon 3a^10^ showed that they were identical, and that both *An. dirus* and *An. minimus* polypeptide sequences were identical with that of *An. gambiae* (Fig. 2). The *Anopheles* GluCl protein sequence showed 42% identity with that of *C. elegans* GluCl (Accession No. NP_507090.1). The only difference between *C. elegans* and *Anopheles* M2-M3 loops is an ILE273 to THR304 substitution (Fig. 2).

### In silico docking model

**Fig. 3:**
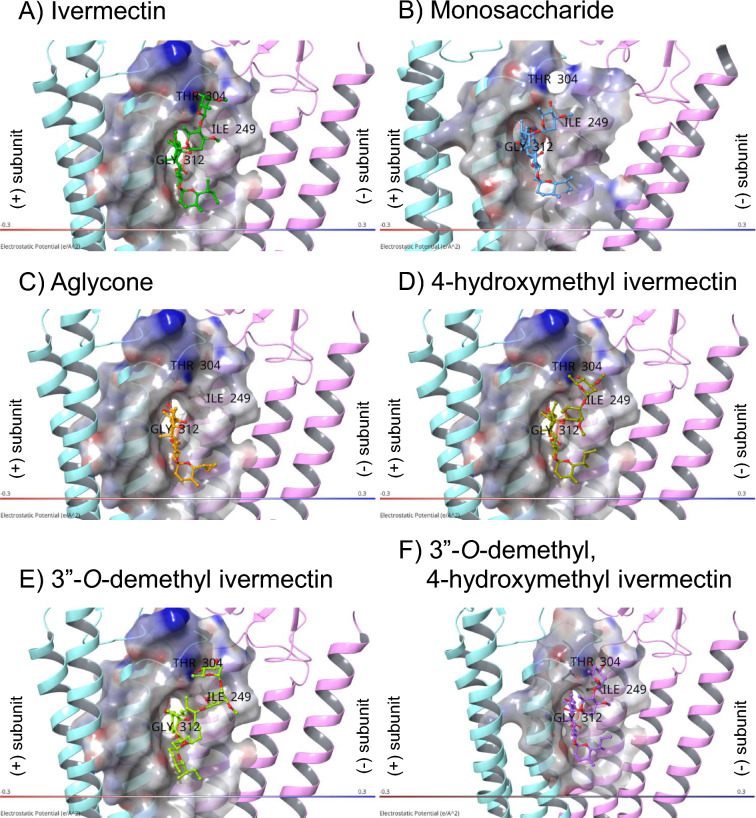
AnGluCl docking models depicting the orientation of ivermectin (**A**), monosaccharide (**B**), aglycone (**C**), 4-hydroxymethyl ivermectin (**D**), 3″-*O*-demethyl ivermectin (**E**), and 3″-*O*-demethyl, 4-hydroxymethyl ivermectin (**F**) within the AnGluCl channel. ( +) subunit M2-M3 loop THR304 is labeled. The AnGluCl equivalent of key binding residues from CeGluCl are labeled as follows: AnGluCl ( +) subunit M3 GLY312 which is equivalent to CeGluCl GLY281 and AnGluCl (-) subunit M1 ILE249 which is equivalent to CeGluCl LEU 218.

*Anopheles* GluCl (AnGluCl) docking models for ivermectin, monosaccharide, aglycone, and metabolites are depicted in Fig. 3. It can be observed that ivermectin and all three of its metabolites extend further into the GluCl channel, with their second sugar ring positioned closer to THR304 of the ( +) subunit M2–M3 loop, compared to either the monosaccharide or aglycone. While hydroxylation at the C-4 position in 4-hydroxymethyl ivermectin and 3″-*O*-demethyl, 4-hydroxymethyl ivermectin contributes to a local rotation of the lactone ring, demethylation at the 3″-*O* position in 3″-*O*-demethyl ivermectin also induces a distinct binding orientation through loss of methoxy-mediated hydrophobic contacts, resulting in a shift of the macrolide backbone relative to the ivermectin pose. Although the three ivermectin metabolites overall adopt binding poses similar to that of ivermectin, the hydroxylation at the C-4 and demethylation-induced reorientation together lead to subtle conformational changes that may influence their interaction networks within the binding pocket and potentially alter binding affinity or functional modulation of the channel.

### Ivermectin

**Fig. 4:**
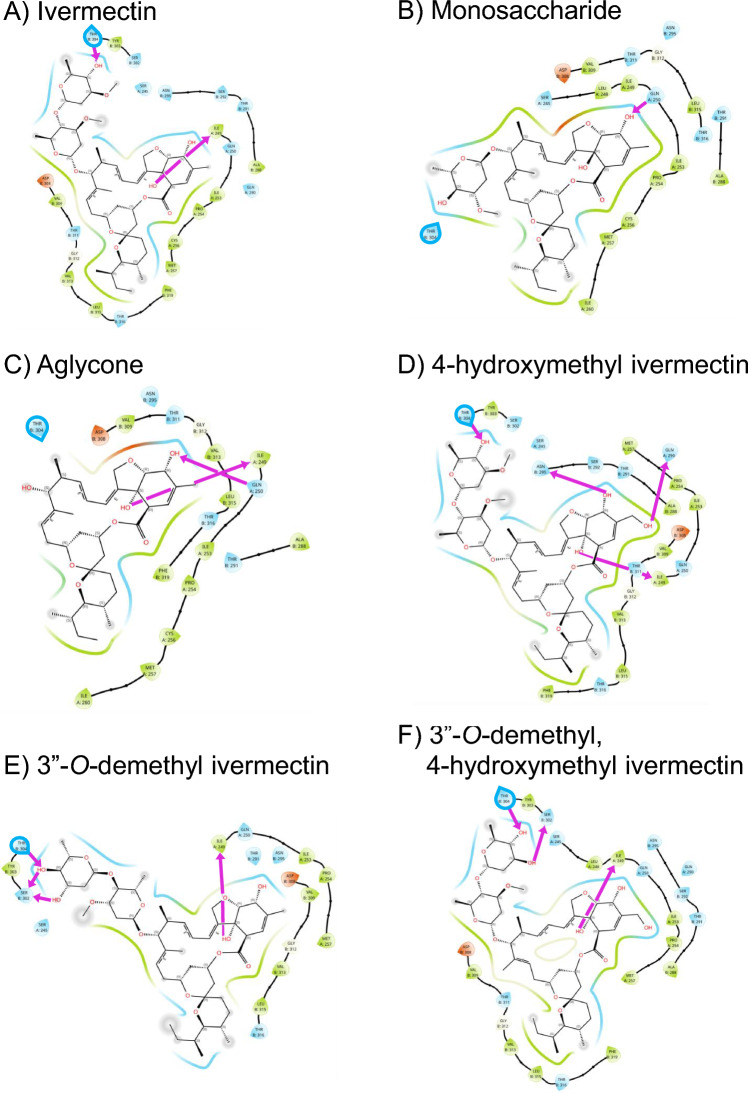
*Anopheles* GluCl docking models and their molecular interactions with ivermectin (**A**), monosaccharide (**B**), aglycone (**C**), 4-hydroxymethyl ivermectin (**D**), 3″-*O*-demethyl ivermectin (**E**), and 3″-*O*-demethyl, 4-hydroxymethyl ivermectin (**F**). AnGluCl amino acid residues in the binding pocket are represented as teardrop shapes and color-coded based on physicochemical properties: green (hydrophobic), blue (polar), orange (negatively charged), and white (glycine). Residues on the (–) subunit are labeled with an “A”, while those on the ( +) subunit are labeled with a “B”. Hydrogen bonds between the ligand and protein are indicated by purple directional arrows. THR304 is outlined in blue. Van der Waals (VDW) interactions are illustrated as colored surface bands surrounding the ligand: green for hydrophobic contacts, blue for polar interactions, and orange for interactions with negatively charged residues. These visual encodings facilitate the interpretation of ligand-residue interaction patterns within the binding pocket.

Ivermectin (Fig. 3A) has a docking score of -9.581 kcal/mol with AnGluCl. It forms a hydrogen bond between the cyclohexene C-7 hydroxyl group and the mainchain oxygen atom of ILE249 in the (-) subunit M1 of AnGluCl, which is equivalent to LEU218 in the (-) subunit M1 of CeGluCl. There is a previously unidentified hydrogen bond between the second sugar ring (4″-OH) and the ( +) subunit M2-M3 loop (THR304) which was identified by the in silico docking model, forming a hydrogen bond between the C-4'' hydroxyl group (4″-OH) and the mainchain nitrogen atom of THR304. Additional VDW interactions at ( +) subunit M2-M3 loop (SER302, TYR303) with the second sugar ring were identified from the docking model (Table 2). Ivermectin spiroketal forms VDW interactions to GLY312 in the ( +) subunit M3 of AnGluCl, which is equivalent to GLY281 in the ( +) subunit M3 of CeGluCl (Table 2; Fig. 4A). In contrast to CeGluCl, no VDW interactions were observed with the AnGluCl M2-M3 loop and the first sugar ring or spiroketal portions (Fig. 4A). This suggests that the mosquito-lethal effect is facilitated by the second sugar ring 4″-OH hydrogen bond with M2-M3 loop THR304, and VDW interactions with SER302 and TYR303.

**Table 2:** Summary of interactions of ivermectin, monosaccharide, aglycone, 4-hydroxymethyl ivermectin (4-OH IVM), 3″-*O*-demethyl ivermectin (3″-*O* IVM), 3″-*O*-demethyl, 4-hydroxymethyl ivermectin (3″-*O*, 4-OH IVM) with AnGluCl amino acids including hydrogen bonds (H-bonds) and Van Der Waals (VDW) forces.

	Ivermectin	Monosaccharide	Aglycone	4-OH IVM	3″-*O* IVM	3″-*O*, 4-OH IVM
Docking score (kcal/mol)	-9.581	-6.728	-7.748	-9.374	-6.347	-10.961
ILE249 (- M1)	H-bond (2.58 Å)	-	H-bond (2.83 Å)	H-bond (2.65 Å)	H-bond (3.24 Å)	H-bond (2.65 Å)
GLN250 (- M1)	-	H-bond (3.17 Å)	H-bond (3.03 Å)	-	-	-
GLN290 (- M1)	-	-	-	H-bond (3.59 Å)	-	-
ASN295 (+ M2)	-	-	-	H-bond (3.04 Å)	-	-
SER302 (+ M2-M3)	VDW	-	-	VDW	H-bond (2.90 Å)	H-bond (2.89 Å)
TYR303 (+ M2-M3)	VDW	-	-	VDW	VDW	VDW
THR304 (+ M2-M3)	H-bond (3.08 Å)	VDW	-	H-bond (2.85 Å)	H-bond (3.01 Å)	H-bond (2.86 Å)
GLY312 (+ M3)	VDW	-	VDW	VDW	VDW	VDW
LEU315 (+ M3)	VDW	-	-	VDW	VDW	VDW

Below each amino acid states its location on the subunit (-, +), transmembrane domain (M1, M2, M3), or extracellular M2-M3 loop. H-bonding distances (in Å) extracted from the docking output have been added to complement the docking scores.

### Monosaccharide

**Fig. 5:**
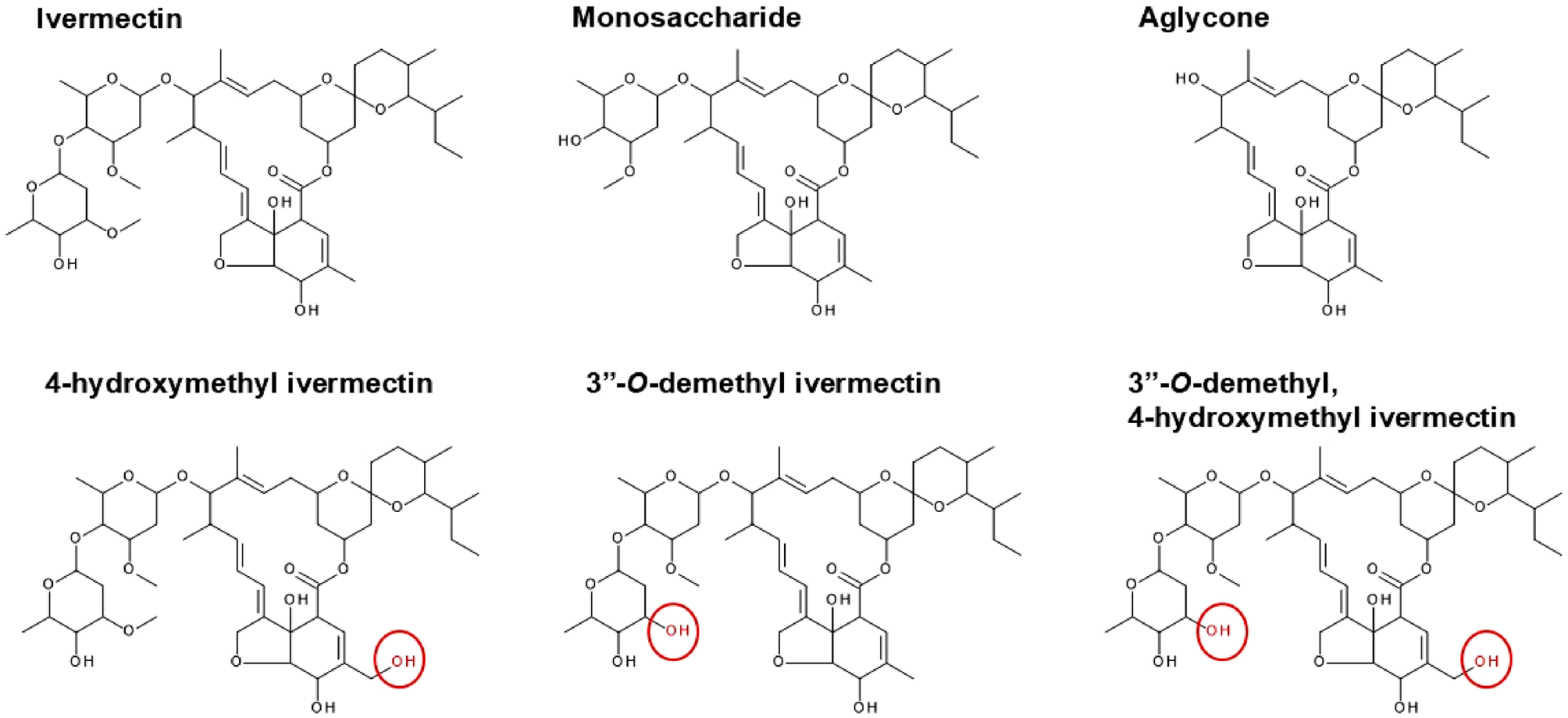
Molecular structure of ivermectin, monosaccharide, aglycone, and metabolites. Ivermectin monosaccharide is missing the second saccharide sugar ring, and aglycone is missing both saccharide sugar rings at C-13. The demethylation and hydroxylation sites of the ivermectin metabolites are circled and colored in red.

Monosaccharide has a docking score of -6.728 kcal/mol with AnGluCl. For monosaccharide (Fig. 3B), the binding conformation is twisted slightly compared to ivermectin. Monosaccharide only forms a hydrogen bond between C-5 hydroxyl group of the cyclohexene cyclic ether unit bound to GLN250 in the (-) subunit M1 of AnGluCl. In contrast to ivermectin, no hydrogen bonds are formed with ( +) subunit M2-M3 loop and there is only one residue (THR304) with VDW interaction to the sugar ring of monosaccharide. Monosaccharide cyclohexene forms no VDW interactions to GLY312 in the ( +) subunit M3 of AnGluCl (Fig. 4B). Monosaccharide has an approximately 100-fold reduced mosquito-lethal effect compared to ivermectin (Table 1). We postulate that this is primarily caused by the lack of the second sugar ring (Fig. 5) extending further into the GluCl channel to form a hydrogen bond with 4″-OH and THR304 of the ( +) subunit M2-M3 loop. However, there is a weaker VDW bond formed with THR304 (Table 2; Fig. 4B) which may facilitate the partial mosquito-lethal effect (Table 1).

### Aglycone

Aglycone has a docking score of -7.741 kcal/mol with AnGluCl. For aglycone (Fig. 3C), the binding conformation is rotated compared to ivermectin. Aglycone forms two hydrogen bonds with the (-) subunit M1 between the cyclohexene C-7 hydroxyl group and the mainchain oxygen atom of ILE249 between C-5 hydroxyl group of the cyclohexene cyclic ether unit bound to GLN250 in the (-) subunit M1. In contrast to ivermectin, no hydrogen bonds or VDW are formed with ( +) subunit M2-M3 loop (Table 2, Fig. 4C). This lack of interaction with the ( +) subunit M2-M3 loop likely explains the lack of mosquito-lethal effect observed with aglycone (Table 1). Aglycone cyclohexene forms VDW interactions to GLY312 in the ( +) subunit M3 of AnGluCl (Fig. 4C).

### 4-hydroxymethyl ivermectin

4-hydroxyl ivermectin has a docking score of -9.374 kcal/mol with AnGluCl. For 4-hydroxyl ivermectin (Fig. 3D), the binding conformation is substantially rotated compared to ivermectin. 4-hydroxyl ivermectin forms two hydrogen bonds with the (-) subunit M1 helix: one between the C7 hydroxyl group on the cyclohexene ring and the mainchain oxygen atom of ILE249, and a second hydrogen bond between the C-4 hydroxyl group and the side chain OE1 atom of GLN290. These interactions may contribute to the altered binding orientation and enhanced stabilization of the 4-hydroxyl ivermectin within the AnGluCl channel. There is an additional hydrogen bond between the C-5 hydroxyl group of the cyclohexene cyclic ether unit bound to ASN295 in the ( +) subunit M2, which is equivalent to ASN264 in the ( +) subunit M2 of CeGluCl. Similar to ivermectin, there is a hydrogen bond formed with the second sugar ring (4″-OH) to the ( +) subunit M2-M3 loop (THR304) and additional VDW interactions at ( +) subunit M2-M3 loop (SER302, TYR303). 4-hydroxyl ivermectin lactone forms VDW interactions to GLY312 in the ( +) subunit M3 of AnGluCl (Fig. 4D). Similar to ivermectin, no VDW interactions were observed with the AnGluCl M2-M3 loop and the first sugar ring or spiroketal portions (Fig. 4D). This suggests that the ivermectin equivalent mosquito-lethal effect^11^ is facilitated by the second sugar ring 4″-OH hydrogen bond with M2-M3 loop THR304, and VDW interactions with SER302 and TYR303.

### 3″-*O*-demethyl ivermectin

3″-*O*-demethyl ivermectin has a docking score of -6.347 kcal/mol with AnGluCl. The two sugar rings of 3″-*O*-demethyl ivermectin (Fig. 4E) are completely rotated ~ 180° compared to ivermectin and the other two metabolites. Notably, three hydrogen bonds were formed between the second sugar ring and the ( +) subunit M2–M3 loop. These include hydrogen bonds between the C-3″ and C-4″ hydroxyl groups and the mainchain oxygen atom of SER302 and the side chain OG1 atom of THR304, respectively. An additional hydrogen bond is formed between the C-4″ hydroxyl group and the mainchain oxygen atom of SER302. These interactions may contribute to the distinct binding mode. VDW interaction at ( +) subunit M2-M3 loop (TYR303) with the second sugar ring was identified from the docking model. 3″-*O*-demethyl ivermectin forms a hydrogen bond between the cyclohexene C-7 hydroxyl group and the mainchain oxygen atom of ILE249 in the (-) subunit M1. 3″-*O*-demethyl ivermectin cyclohexene forms VDW interactions to GLY312 in the ( +) subunit M3 of AnGluCl (Fig. 4E). Although 3″-*O*-demethyl ivermectin forms additional hydrogen bonds, its overall docking score decreases due to unfavorable contributions from other energetic terms. These include increased ligand strain and suboptimal hydrogen bond geometry, as the ligand may need to twist, bend, or stretch its bonds to form hydrogen bonds or to fit into the binding pocket. This likely contributes to the markedly different orientation of its two sugar rings compared to those in ivermectin and the other two metabolites. Similar to ivermectin, no VDW interactions were observed with the AnGluCl M2-M3 loop and the first sugar ring or spiroketal portions (Fig. 4E). This suggests that the ivermectin equivalent mosquito-lethal effect^11^ is facilitated by the second sugar ring 3″-OH and 4″-OH hydrogen bonds with ( +) subunit M2-M3 loop THR304 and SER302, and VDW interactions with TYR303.

### 3″-*O*-demethyl, 4-hydroxymethyl ivermectin

3″-*O*-demethyl, 4-hydroxymethyl ivermectin exhibits a more favorable docking score of − 10.961 kcal/mol compared to ivermectin, suggesting stronger predicted binding to AnGluCl. Its binding conformation differs notably from ivermectin with a rotated orientation of the second sugar ring, enabling additional hydrogen bond with residues in the ( +) subunit M2–M3 loop (Fig. 4F). Two hydrogen bonds were formed between the second sugar ring and the ( +) subunit M2–M3 loop between the C-3″ and C-4'' hydroxyl groups and the mainchain oxygen atom of SER 302 and the mainchain nitrogen atom of THR304, respectively. 3″-*O*-demethyl, 4-hydroxymethyl ivermectin forms a hydrogen bond between the cyclohexene C-7 hydroxyl group and the mainchain oxygen atom of ILE249 in the (-) subunit M1 of AnGluCl. Additional VDW interaction at ( +) subunit M2-M3 loop (TYR303) with the second sugar ring was identified from the docking model. 3″-*O*-demethyl ivermectin spiroketal forms VDW interactions to GLY312 in the ( +) subunit M3 of AnGluCl (Fig. 4F). Similar to ivermectin, no VDW interactions were observed with the AnGluCl M2-M3 loop and the first sugar ring or spiroketal portions (Fig. 4F). This suggests that the ivermectin equivalent mosquito-lethal effect^11^ is facilitated by the second sugar ring 4″-OH and 3″-OH hydrogen bonds with ( +) subunit M2-M3 loop THR304 and SER302, and VDW interactions with TYR303.

Stereochemical details of ivermectin and its analogs are displayed in Supplemental Figure S3. Images were generated using Schrödinger Maestro, with chiral centers indicated by wedge and dashed bonds.

## Discussion

In this study, we have identified additional hydrogen bonds formed between the ( +) subunit M2-M3 loop of *Anopheles* GluCl and the second sugar ring (4″-OH) of ivermectin (Fig. 4A) and all three metabolites (Figs. 4D-F) from our docking models. These binding sites form hydrogen bonds between the C-4’’ hydroxyl group (4″-OH) of the second sugar ring in the disaccharide unit with the mainchain nitrogen atom of THR304 in the ( +) subunit M2-M3 loop (Fig. 4, Table 2). This is possible due to a single amino acid change from CeGluCl ILE273 to AnGluCl THR304 (Fig. 2), wherein the hydrogen of the THR304 hydroxyl group acts as a hydrogen bond donor to the oxygen atom on the second sugar ring (4’’-OH) (Supplemental Figure S1B), potentially stabilizing the loop and restricting its flexibility. Newly identified VDW interactions are found between the second sugar ring of ivermectin (Fig. 4A) and residues SER302 and TYR303 in the ( +) subunit M2-M3 loop. In *Drosophila melanogaster*, a single amino acid substitution in the DmGluCl M2-M3 loop from PRO299 to SER299 caused an observed 3.3-fold decrease in ivermectin lethal effect indicating that the M2-M3 loop is important for ivermectin activity in other Diptera as well^5^. Since the M2-M3 loop is identical in DmGluCl and AnGluCl, this suggests that site mutations in any amino acids found in the M2-M3 loop could lead to potential ivermectin resistance in *Anopheles*.

Ivermectin monosaccharide metabolites were produced in pooled human liver microsomes^13^ and identified from human blood samples^12^. Monosaccharide had intermediate mosquito-lethal effect on *An. dirus* and *An. minimus*, compared to ivermectin with an approximately 111-fold and 83-fold reduction, respectively (Table 1; Fig. 1). Only forming VDW to ( +) subunit M2-M3 loop THR304 (Fig. 4B) may have caused this intermediate mosquito-lethal effect. This suggests that the hydrogen bond between the C-4’’ hydroxyl group (OH) of the second sugar ring in the disaccharide unit of ivermectin and the mainchain nitrogen atom of THR304 in the ( +) subunit M2-M3 loop is critical for *Anopheles* GluCl channel activation. Compared to ivermectin, monosaccharide induced the same level of mortality against the parasitic ruminant nematode, *Haemonchus contortus*^14^, supporting the proposed *C. elegans* model that VDW interactions between the first sugar ring and spiroketal (Supplemental Figure S1A) with the M2-M3 loop is critical for activation of the GluCl channel in nematodes^2^. In contrast to CeGluCl, there were no VDW interactions with the AnGluCl M2-M3 loop and the first sugar ring or spiroketal for ivermectin, monosaccharide, or metabolites (Table 2; Fig. 4), indicating a potential major departure in how AnGluCl interacts with ivermectin from the previous CeGluCl model^2^.

Consistent across ivermectin, aglycone, and all metabolites was the hydrogen bond between the cyclohexene C-7 hydroxyl group and the AnGluCl (-) subunit M1 ILE249 which corresponds to CeGluCl (-) subunit M1 LEU218, which was the only bond to the (-) subunit identified from *C. elegans*, indicating that this interaction is critical to ivermectin activity. Interestingly, both monosaccharide and aglycone form a hydrogen bond between the cyclohexene C-5 hydroxyl group and GLN250 on the AnGluCl (-) subunit M1 (Table 2, Figs. 4B, 4C). This distinct interaction likely alters their positioning within the channel compared to ivermectin, potentially leading to weaker engagement with gating-related residues and thus reducing their mosquito-lethal activity. This suggests that an AnGluCl ILE249 target site mutation may not lead to total loss of function in *Anopheles* if ivermectin could bind to nearby residues, but this possibility needs to be explored in functional assays.

Aglycone was not detected in human liver microsomes, hepatocytes, or blood samples ^12,13^. Aglycone lacks interaction with AnGluCl M2-M3 loop (Figs. 3C, 4C) and has no mosquito-lethal effect on *An. dirus*, *An. minimus* (Table 1; Fig. 1), or *Anopheles stephensi*^15^. Conversely, aglycone was found to activate the CeGluClα channel, although with less efficacy than ivermectin^16^, which suggests that the hydrogen bonds to ( +) subunit M2 or M3 are important for GluCl channel activation in *C. elegans*. Indeed, the cyclohexene C-5 hydrogen bond to SER260 in the ( +) subunit M2 has been shown to be critical in *C. elegans*^16^ and alterations to the C-5 hydroxyl group ablates efficacy against *H. contortus*^14^, however it could be possible for the C-5 hydroxyl group to bind with other residues on ( +) subunit M2 so the importance of SER260 for the M2 binding is in question^17^. For *Anopheles*, no hydrogen bonds were found between ivermectin and AnGluCl ( +) subunit M2, which could be due to a CeGluCl SER260 to AnGluCl THR291 change. The bulkier threonine side chain may introduce steric hindrance or alter hydrogen bond geometry, reducing interaction potential. Alternatively, the positioning of ivermectin within the AnGluCl channel may prevent optimal alignment for hydrogen bonding at this site. The 4-hydroxyl ivermectin forms a hydrogen bond between C-5 hydroxyl group with the AnGluCl ( +) subunit M2 ASN295 (Fig. 4D), but since this metabolite expresses equal mosquito-lethal effect as ivermectin^11^ it does not appear that binding to ( +) subunit M2 is necessary nor would it improve ivermectin mosquito-lethal effect.

In *C. elegans* a hydrogen bond forms between ( +) subunit M3 THR285 and the ivermectin spiroketal O-14. However, no hydrogen bonds were observed with any structures and the AnGluCl ( +) subunit M3 THR316 equivalent, in fact no hydrogen bonds were predicted with AnGluCl M3 at all. In *Musca domestica*, a THR316 to VAL316 substitution produced no change in GluCl activation by ivermectin^18^, suggesting that a hydrogen bond with GluCl ( +) subunit M3 THR316 is not necessary for channel activation in Diptera. While the THR316 to VAL316 substitution abolishes hydrogen bonding capability due to the absence of a hydroxyl group, it retains potential for VDW interactions. However, in nematode models, the formation of VDW with ( +) subunit M3 GLY281 appears to be critical for gating interactions in the GluCl channel^19^. Interestingly, in *Anopheles*, *D. melanogaster*, and *M. domestica* the ( +) subunit M3 GLY312 (*C. elegans* GLY281 equivalent) is highly conserved and substitution experiments demonstrated reduced GluCl channel activity when GLY312 was replaced with MET312^20^. Furthermore, in *M. domestica*, changes to ( +) subunit M3 LEU315 to PHE315, TYR315, or HIS315 dramatically reduced GluCl activation^18^. Taken together, these points suggest that in *Anopheles* and other Diptera, hydrogen bonds with ( +) subunit M3 THR316 may not be essential for GluCl channel activation, but there may be critical VDW interactions with nearby residues along M3 (*e.g.* GLY312 or LEU315) which may gate GluCl activity. Since monosaccharide does not form VDW with AnGluCl ( +) subunit M3 GLY312, this may also contribute to the partial mosquito-lethal effect of monosaccharide. Excluding monosaccharide, all other structures can form VDW with AnGluCl ( +) subunit M3 GLY312 (Table 2) but due to rotation of the structures within the AnGluCl channel (Fig. 3) none are in exactly the same position with the spiroketal region of ivermectin (Fig. 4A). Further experiments are required to elucidate the contribution of ( +) subunit M3 VDW interactions with AnGluCl on mosquito-lethal effect.

In *C. elegans* the cyclohexene C-4 methyl group forms VDW bonds to CeGluCl ( +) subunit M2 THR257. Both the 4-hydroxyl ivermectin and 3″-*O*-demethyl, 4-hydroxymethyl ivermectin have a hydroxylation at cyclohexene C-4. The AnGluCl docking models predicts this cyclohexene C-4 hydroxyl group will form a hydrogen bond with the (-) subunit M1 GLN290 for 4-hydroxyl ivermectin (Fig. 4D) but for 3″-*O*-demethyl, 4-hydroxymethyl ivermectin will form VDW with (-) subunit M1 (Fig. 4F). Interestingly, the AnGluCl docking models do not predict ivermectin (Fig. 4A) nor 3″-*O*-demethyl ivermectin (Fig. 4E) C-4 methyl group will form VDW with the GluCl channel.

The demethylation of C-3″ on the second sugar ring observed with 3″-*O*-demethyl ivermectin and 3″-*O*-demethyl, 4-hydroxymethyl ivermectin allows for the formation of additional hydrogen bonds with ( +) subunit M2-M3 loop SER302 (Figs. 4E, F). For 3″-*O*-demethyl ivermectin three hydrogen bonds were formed between THR304 and SER302 and the C-3″ and C-4″ hydroxyl groups (Fig. 4E) but only two hydrogen bonds were formed with 3″-*O*-demethyl, 4-hydroxymethyl ivermectin (Fig. 4F). While this difference may be partly attributed to the ~ 180° rotation of the sugar rings in 3″-*O*-demethyl ivermectin (Fig. 4E), other factors such as steric hindrance from the additional hydroxymethyl group, altered ligand flexibility, or changes in intramolecular hydrogen bonding may also limit hydrogen bond formation with nearby residues. Although 3″-*O*-demethyl ivermectin and 3″-*O*-demethyl, 4-hydroxymethyl ivermectin demonstrate comparable mosquito-lethal activity to ivermectin^11^, the lower docking score of 3″-*O*-demethyl ivermectin suggests that further structural modifications may not significantly enhance binding affinity to AnGluCl. This could indicate that ivermectin and its active metabolites already achieve near-maximal engagement with key binding regions such as the M2–M3 loop. Nevertheless, the formation of additional hydrogen bonds with M2–M3 loop residues may still play a role in receptor subtype specificity or binding dynamics not fully captured by static docking analyses.

This work deepens our understanding regarding how ivermectin binds to *Anopheles* GluCl and for potential future mechanisms of ivermectin resistance development. The basis for the varying tolerance of different *Anopheles* species to ivermectin has yet to be explained. There are no striking differences in their GluCl protein sequences that may offer an obvious explanation for differential actions of ivermectin, as the amino acid sequences of *An. dirus*, *An. gambiae*, and *An. minimus* GluCls with exon 3a are identical. The other two exon 3 splice variants (3b and 3c) in *An. dirus* differ from those of *An. gambiae* and *An. minimus* by a single amino acid (Supplemental Figure S2). This introduces variation in the extracellular N-terminal region, which was not implicated as the binding site of ivermectin. In line with this, heterologous expression of *An. gambiae* GluCl in *Xenopus laevis* oocytes showed that exon 3b and 3c variants showed similar sensitivity to ivermectin^21^. However, splicing of *Bombyx mori* GluCl exon 3 was shown to affect the size of ivermectin-induced responses, most likely through altering cell surface expression of the receptor^22^. In addition, alternative splicing has been shown to alter the length of the large intracellular M3-M4 loop of *Anopheles* GluCl^10^. Since studies have shown that different M3-M4 loop variants can affect the sensitivity of the closely related γ-aminobutyric acid (GABA) channel to fipronil^23^, it will be of interest to see whether altered expression of GluCl splice variants may contribute to species-specific sensitivity to ivermectin. In this regard, it is interesting to note that the presence of certain splice variants of *Chilo suppressalis* GluCl was found to be associated with resistance to emamectin benzoate^24^. RNA A-to-I editing is another mechanism known to introduce variation in insect GluCl^25,26^ thus it is worth studying the presence and extent of RNA editing of GluCl in the different *Anopheles* species. Whilst GluCl is considered the primary target of ivermectin^6^, other ion channels such as the GABA receptor, RDL (resistance to dieldrin), may be secondary targets^27^. With the finding that RNA editing of the *Anopheles* RDL or the A301S (resistance to dieldrin) mutation can affect the actions of ivermectin^28,29^, it may prove instructive to characterize *Rdl* variants^30^ in *An. dirus* and *An. minimus*. In investigating the differential tolerance of the *Anopheles* species to ivermectin, it is also prudent to consider other mechanisms of resistance other than the GluCl target site, such as detoxification mechanisms mediated by cytochrome P450 enzymes as observed in *Aedes aegypti*^31^.

 The primary limitation of this study is a lack of functional assay evaluation to verify the relevance of the proposed AnGluCl-ivermectin binding interactions. Validation of the proposed 3-D docking conformations of AnGluCl-ivermectin binding with Cryo-Electron Microscopy would be beneficial for testing the interactions proposed here and possibly identifying additional interactions. Overall, we propose a novel conformation of ivermectin-GluCl interactions in *Anopheles* wherein ivermectin binds via hydrogen bonds only to the ( +) subunit M2-M3 loop and (-) subunit M1 and likely with VDW to the ( +) subunit M3, and this potentially extends to other Diptera. In conclusion, we propose additional *Anopheles* GluCl-ivermectin interactions not identified in the CeGluCl-ivermectin model, such as the hydrogen bond with the second ring C-4″ with the M2-M3 loop THR304, which may be critical for channel activation and mosquito-lethal effect.

## Methods

### Compounds

Powdered formulations of ivermectin were obtained from Sigma-Aldrich (St. Louis, Missouri, USA), monosaccharide from ClearSynth (Mississauga, Ontario, Canada) and aglycone (Cayman Chemical, Ann Arbor, Michigan, USA). Ivermectin metabolites were acquired 4-hydroxymethyl ivermectin (WuXi AppTec (Tianjin) Co., Ltd, Tianjin, China) or produced 3″-*O*-demethyl ivermectin and 3″-*O*-demethyl, 4-hydroxymethyl ivermectin (Hypha Discovery, Abingdon, UK) as described previously^11^ (Fig. 5). Compounds were dissolved in dimethylsulfoxide (DMSO) to concentrations of 2 mg/ml and 12 μl aliquots were frozen at − 20 °C.

### Mosquitoes

All mosquitoes were reared at the Insectary of the Department of Medical Entomology, Faculty of Tropical Medicine, Mahidol University in Bangkok, Thailand. *Anopheles dirus* s.s. and *Anopheles minimus* s.s. were produced as described previously^32^. Adult mosquitoes used for experiments were provided 5% multivitamin syrup with 5% sucrose solution (Seven Seas, PT. Merck Tbk., Jakarta Indonesia) for the first 48 h post emergence and then 10% sucrose solution ad libitum. Female mosquitoes were between 5- and 7-days post emergence at time of blood feeding, and were sugar-starved with access to water from 16 to 18 h before their blood meal.

### Mosquito blood meal preparation

Mosquito blood meal preparation was performed as described previously^32^. Whole blood was collected from healthy volunteers on the day of each mosquito membrane feed. Blood was drawn into sodium heparin tubes. Compounds were thawed and serial dilutions were made in human AB + plasma and glass amber vials with 10 μl added to 990 μl of whole blood to reach final concentration desired for mosquito membrane feeding assays. Control blood meals consisted of previously frozen DMSO diluted in plasma to match the highest ratio of DMSO and plasma fed to mosquitoes in the compound-containing blood meals.

### Mosquito membrane feeding and mortality assays

Mosquito membrane feeding, mortality assays and analyses were performed as described previously^32^. At each mosquito membrane feed approximately 1 ml of whole blood mixed with the different compounds over a range of concentrations were offered to groups of 40 *An. dirus* and 40 *An. minimus* mosquitoes via membrane feeders warmed to 37 °C. After feeding, up to 30 blood-fed mosquitoes of each species were gently transferred via aspiration to clean cardboard containers (0.5 L). After the blood meal, mosquitoes were maintained in an incubator at 25 ± 1 °C and 80 ± 10% humidity, and offered 10% sucrose ad libitum. Mosquito survival was monitored daily for ten days and any dead mosquitoes were removed by aspiration and recorded. Ten days after the blood meal any remaining mosquitoes were recorded as alive and then frozen. The lethal concentration that kills 50% (LC_50_) of mosquitoes were estimated using a normalized concentration–response analysis (IC_50_ and Hill), assuming a maximum of 100% mosquito mortality and an estimated

baseline mosquito mortality (*i.e.* mosquito mortality at zero drug concentration). All mosquito survival analyses were performed with GraphPad Prism v.10.2 (GraphPad Software Inc, San Diego, CA, USA).

### Identification and amplification of *An. dirus* and *An. minimus* GluCl

Total RNA was extracted from groups of ten adult female *An. dirus* and *An. minimus* of each species using Trizol following the manufacturer’s protocol (Thermo Fisher Scientific, Loughborough, UK). First-strand cDNA was synthesized from 1 μg of total RNA using GoScript™ Reverse Transcriptase (Promega, Southampton, UK). Putative exon sequences encoding *An. dirus* and *An. minimus* GluCl subunits were identified by screening their respective genome sequences available at NCBI (https://blast.ncbi.nlm.nih.gov/Blast.cgi) using the tBLASTn algorithm^33^ with the *An. gambiae* GluCl sequence (Accession No. XP_061519550.1). This sequence information was used to design forward and reverse primers (Supplemental Table S1) to amplify the whole cDNA coding sequences of *An. dirus* or *An. minimus* GluCl using Q5 High Fidelity DNA Polymerase (New England Biolabs, Hitchin, UK). Amplification products were sequenced by SourceBioScience (https://genomics.sourcebioscience.com/). The *An. dirus* or *An. minimus* GluCl cDNA sequences have been deposited in GenBank with accession numbers PV568088 and PV568089, respectively.

### Analysis of Anopheles GluCl peptide sequences

The alignment of the *Anopheles* and *C. elegans* GluCl sequences were constructed using Clustal X with default settings^34^. The protein alignment was viewed using GeneDoc (http://www.nrbsc.org/gfx/genedoc/index.html), which was also used to calculate identity values.

### In silico docking methods

In this study, the new AI technique of AlphaFold 3.0 (https://alphafoldserver.com/) was first used to predict the full-length dimeric structure of AnGluCl with the predicted template modeling (pTM) score = 0.66 and the interface predicted template modeling (ipTM) score = 0.61. The AlphaFold 3.0 model achieved moderately good scores of pTM and ipTM, suggesting reliable 3D structure prediction of *Anopheles* GluCl. After that our CLICK method (http://cospi.iiserpune.ac.in/click/) was utilized to identify the binding pocket of ivermectin on the AlphaFold 3.0 model of AnGluCl. The Schrodinger software Release 2025–2; the Glide Extra precision (XP) mode (https://www.schrodinger.com/) was then employed to dock ivermectin, monosaccharide, aglycone, and the metabolites (3″-*O*-demethyl ivermectin, 4-hydroxymethyl ivermectin, and 3″-*O*-demethyl, 4-hydroxymethyl ivermectin) with the AlphaFold 3.0 model of AnGluCl based on the binding pockets identified by CLICK. The CLICK method performs 3D structural superposition of molecular pairs based on the similarity of local structural packing, enabling the alignment of structures with dissimilar topologies, conformations, or even molecular types^35–40^. These unique capabilities make CLICK ideally suited for comparing similarities between binding pockets.

Ivermectin, monosaccharide, aglycone, and metabolite derivatives, are macrocyclic lactones featuring large and flexible ring systems. These structural characteristics present challenges for conventional molecular docking methods, due to their complex conformational landscapes. To address this, the “Sample macrocycles using Prime” option within Schrödinger’s Glide docking tool was applied. This approach enables more accurate sampling for conformational space of ivermectin, monosaccharide, aglycone, and the metabolites by generating low-energy, ring-closed structures. The Prime method applies advanced ring sampling algorithms that maintain realistic torsional angles and internal strain during conformer generation. As a result, the docking accuracies of ivermectin, monosaccharide, aglycone, and the metabolites to the AnGluCl binding pocket are improved, providing conformations that more effectively complement the geometry of the protein binding site.

Finally, the docking analyses were performed to identify key binding residues of AnGluCl as well as the interacting atoms of ivermectin, monosaccharide, aglycone, and metabolite derivatives in order to characterize critical interactions and evaluate their corresponding Glide docking scores.

## Supplementary Information

Supplementary Information.

## Data Availability

Data is provided within the manuscript or supplementary information files.

## References

[1] Billingsley, P. et al. A Roadmap for the Development of Ivermectin as a Complementary Malaria Vector Control Tool. *Am. J. Trop. Med. Hyg.* **102**, 3–24. 10.4269/ajtmh.19-0620 (2020). 31971144 10.4269/ajtmh.19-0620 PMC7008306

[2] Hibbs, R. & Gouaux, E. Principles of activation and permeation in an anion-selective Cys-loop receptor. *Nature* **474**, 54–60. 10.1038/nature10139 (2011). 21572436 10.1038/nature10139 PMC3160419

[3] Cully, D. et al. Cloning of an avermectin-sensitive glutamate-gated chloride channel from *Caenorhabditis elegans*. *Nature* **371**, 707–711. 10.1038/371707a0 (1994). 7935817 10.1038/371707a0

[4] Cully, D., Paress, P., Liu, K., Schaeffer, J. & Arena, J. Identification of a *Drosophila melanogaster* glutamate-gated chloride channel sensitive to the antiparasitic agent avermectin. *J. Biol. Chem.* **271**, 20187–20191. 10.1074/jbc.271.33.20187 (1996). 8702744 10.1074/jbc.271.33.20187

[5] Kane, N. et al. Drug-resistant *Drosophila* indicate glutamate-gated chloride channels are targets for the antiparasitics nodulisporic acid and ivermectin. *Proc. Natl. Acad. Sci. USA* **97**, 13949–13954. 10.1073/pnas.240464697 (2000). 11095718 10.1073/pnas.240464697 PMC17681

[6] Wolstenholme, A. Glutamate-gated chloride channels. *J. Biol. Chem.* **287**, 40232–40238. 10.1074/jbc.R112.406280 (2012). 23038250 10.1074/jbc.R112.406280 PMC3504739

[7] Althoff, T., Hibbs, R., Banerjee, S. & Gouaux, E. X-ray structures of GluCl in apo states reveal a gating mechanism of Cys-loop receptors. *Nature* **512**, 333–337. 10.1038/nature13669 (2014). 25143115 10.1038/nature13669 PMC4255919

[8] Jones, A. Genomics, cys-loop ligand-gated ion channels and new targets for the control of insect pests and vectors. *Curr. Opin. Insect Sci.* **30**, 1–7. 10.1016/j.cois.2018.07.016 (2018). 30553480 10.1016/j.cois.2018.07.016

[9] Jones, A. & Sattelle, D. The cys-loop ligand-gated ion channel gene superfamily of the nematode. *Caenorhabditis elegans. Invert. Neurosci.* **8**, 41–47. 10.1007/s10158-008-0068-4 (2008). 18288508 10.1007/s10158-008-0068-4 PMC2257991

[10] Meyers, J. et al. Characterization of the target of ivermectin, the glutamate-gated chloride channel, from *Anopheles gambiae*. *J. Exp. Biol.* **218**, 1478–1486. 10.1242/jeb.118570 (2015). 25994631 10.1242/jeb.118570 PMC4448665

[11] Kobylinski, K. et al. Ivermectin metabolites reduce *Anopheles* survival. *Sci. Rep.* **13**, 8131. 10.1038/s41598-023-34719-2 (2023). 37208382 10.1038/s41598-023-34719-2 PMC10199058

[12] Kern, C. et al. Pharmacokinetics of ivermectin metabolites and their activity against *Anopheles stephensi* mosquitoes. *Malar. J.* **22**, e194. 10.1186/s12936-023-04624-0 (2023). 10.1186/s12936-023-04624-0 PMC10290335 37355605

[13] Tipthara, P. et al. Identification of the metabolites of ivermectin in humans. *Pharmacol. Res. Perspect.* **9**, e00712. 10.1002/prp2.712 (2021). 33497030 10.1002/prp2.712 PMC7836931

[14] Michael, B., Meinke, P. & Shoop, W. Comparison of ivermectin, doramectin, selamectin, and eleven intermediates in a nematode larval development assay. *J. Parasitol.* **87**, 692–696. 10.1645/0022-3395(2001)087[0692:Coidsa]2.0.Co;2 (2001). 11426737 10.1645/0022-3395(2001)087[0692:COIDSA]2.0.CO;2

[15] Singh, L. et al. Molecular Design and Synthesis of Ivermectin Hybrids Targeting Hepatic and Erythrocytic Stages of *Plasmodium* Parasites. *J. Med. Chem.* **63**, 1750–1762. 10.1021/acs.jmedchem.0c00033 (2020). 32011136 10.1021/acs.jmedchem.0c00033

[16] Atif, M. et al. GluClR-mediated inhibitory postsynaptic currents reveal targets for ivermectin and potential mechanisms of ivermectin resistance. *PLoS Pathog.* **15**, e1007570. 10.1371/journal.ppat.1007570 (2019). 30695069 10.1371/journal.ppat.1007570 PMC6368337

[17] Lynagh, T., Webb, T., Dixon, C., Cromer, B. & Lynch, J. Molecular determinants of ivermectin sensitivity at the glycine receptor chloride channel. *J. Biol. Chem.* **286**, 43913–43924. 10.1074/jbc.M111.262634 (2011). 22033924 10.1074/jbc.M111.262634 PMC3243547

[18] Nakata, Y. et al. A Single Amino Acid Substitution in the Third Transmembrane Region Has Opposite Impacts on the Selectivity of the Parasiticides Fluralaner and Ivermectin for Ligand-Gated Chloride Channels. *Mol. Pharmacol.* **92**, 546–555. 10.1124/mol.117.109413 (2017). 28887352 10.1124/mol.117.109413

[19] Lynagh, T. & Lynch, J. Ivermectin binding sites in human and invertebrate Cys-loop receptors. *Trends Pharmacol. Sci.* **33**, 432–441. 10.1016/j.tips.2012.05.002 (2012). 22677714 10.1016/j.tips.2012.05.002

[20] Fuse, T., Kita, T., Nakata, Y., Ozoe, F. & Ozoe, Y. Electrophysiological characterization of ivermectin triple actions on *Musca* chloride channels gated by l-glutamic acid and γ-aminobutyric acid. *Insect Biochem. Mol. Biol.* **77**, 78–86. 10.1016/j.ibmb.2016.08.005 (2016). 27543424 10.1016/j.ibmb.2016.08.005

[21] Atif, M., Lynch, J. & Keramidas, A. The effects of insecticides on two splice variants of the glutamate-gated chloride channel receptor of the major malaria vector. *Anopheles gambiae. Br. J. Pharmacol.* **177**, 175–187. 10.1111/bph.14855 (2020). 31479507 10.1111/bph.14855 PMC6976876

[22] Furutani, S. et al. Exon 3 splicing and mutagenesis identify residues influencing cell surface density of heterologously expressed silkworm (*Bombyx mori*) glutamate-gated chloride channels. *Mol. Pharmacol.* **86**, 686–695. 10.1124/mol.114.095869 (2014). 25261427 10.1124/mol.114.095869

[23] Jiang, F. et al. Identification of polymorphisms in *Cyrtorhinus lividipennis* RDL subunit contributing to fipronil sensitivity. *Pestic. Biochem. Physiol.* **117**, 62–67. 10.1016/j.pestbp.2014.10.010 (2015). 25619913 10.1016/j.pestbp.2014.10.010

[24] Wang, S. et al. Overexpression and alternative splicing of the glutamate-gated chloride channel are associated with emamectin benzoate resistance in the rice stem borer, *Chilo suppressalis* Walker (Lepidoptera: Crambidae). *Pest Manag. Sci.* **81**, 2114–2125. 10.1002/ps.8610 (2025). 39691963 10.1002/ps.8610

[25] Semenov, E. & Pak, W. Diversification of *Drosophila* chloride channel gene by multiple posttranscriptional mRNA modifications. *J. Neurochem.* **72**, 66–72. 10.1046/j.1471-4159.1999.0720066.x (1999). 9886055 10.1046/j.1471-4159.1999.0720066.x

[26] Zak, H. et al. A highly conserved A-to-I RNA editing event within the glutamate-gated chloride channel GluClα is necessary for olfactory-based behaviors in *Drosophila*. *Sci. Adv.* **10**, e9101. 10.1126/sciadv.adi9101 (2024). 10.1126/sciadv.adi9101 PMC11373593 39231215

[27] Wang, Q. et al. Functional analysis reveals ionotropic GABA receptor subunit RDL is a target site of ivermectin and fluralaner in the yellow fever mosquito. *Aedes aegypti. Pest Manag. Sci.* **78**, 4173–4182. 10.1002/ps.7035 (2022). 35690922 10.1002/ps.7035

[28] Taylor-Wells, J., Senan, A., Bermudez, I. & Jones, A. Species specific RNA A-to-I editing of mosquito RDL modulates GABA potency and influences agonistic, potentiating and antagonistic actions of ivermectin. *Insect Biochem. Mol. Biol.* **93**, 1–11. 10.1016/j.ibmb.2017.12.001 (2018). 29223796 10.1016/j.ibmb.2017.12.001

[29] Lees, K. et al. Actions of agonists, fipronil and ivermectin on the predominant in vivo splice and edit variant (RDLbd, I/V) of the Drosophila GABA receptor expressed in *Xenopus laevis* oocytes. *PLoS ONE* **9**, e97468. 10.1371/journal.pone.0097468 (2014). 24823815 10.1371/journal.pone.0097468 PMC4019635

[30] Jones, A. K. How Complex Can Resistance to Dieldrin, the Insect γ-Aminobutyric Acid Receptor, Get?. *Int. J. Insect Sci.* **10**, 1179543318804782. 10.1177/1179543318804782 (2018). 30559597 10.1177/1179543318804782 PMC6291865

[31] Nicolas, P. et al. Potential metabolic resistance mechanisms to ivermectin in *Anopheles gambiae*: a synergist bioassay study. *Parasit. Vectors.* **14**, e172. 10.1186/s13071-021-04675-9 (2021). 10.1186/s13071-021-04675-9 PMC7981804 33743783

[32] Khemrattrakool, P. et al. Impact of ivermectin components on *Anopheles dirus* and *Anopheles minimus* mosquito survival. *Parasit. Vectors.* **17**, e224. 10.1186/s13071-024-06294-6 (2024). 10.1186/s13071-024-06294-6 PMC11097567 38750608

[33] Altschul, S., Gish, W., Miller, W., Myers, E. & Lipman, D. Basic local alignment search tool. *J. Mol. Biol.* **215**, 403–410. 10.1016/s0022-2836(05)80360-2 (1990). 2231712 10.1016/S0022-2836(05)80360-2

[34] Thompson, J., Gibson, T., Plewniak, F., Jeanmougin, F. & Higgins, D. The CLUSTAL_X windows interface: flexible strategies for multiple sequence alignment aided by quality analysis tools. *Nucleic Acids Res.* **25**, 4876–4882. 10.1093/nar/25.24.4876 (1997). 9396791 10.1093/nar/25.24.4876 PMC147148

[35] Nguyen, M., Tan, K. & Madhusudhan, M. CLICK–topology-independent comparison of biomolecular 3D structures. *Nucleic Acids Res.* **39**, W24-28. 10.1093/nar/gkr393 (2011). 21602266 10.1093/nar/gkr393 PMC3125785

[36] Nguyen, M. & Madhusudhan, M. Biological insights from topology independent comparison of protein 3D structures. *Nucleic Acids Res.* **39**, e94. 10.1093/nar/gkr348 (2011). 21596786 10.1093/nar/gkr348 PMC3152366

[37] Nguyen, M., Sim, A., Wan, Y., Madhusudhan, M. & Verma, C. Topology independent comparison of RNA 3D structures using the CLICK algorithm. *Nucleic Acids Res.* **45**, e5. 10.1093/nar/gkw819 (2017). 27634929 10.1093/nar/gkw819 PMC5741206

[38] Nguyen, M. et al. Discovering Putative Protein Targets of Small Molecules: A Study of the p53 Activator Nutlin. *J. Chem. Inf. Model.* **59**, 1529–1546. 10.1021/acs.jcim.8b00762 (2019). 30794402 10.1021/acs.jcim.8b00762

[39] Nguyen, M., Verma, C. & Zhong, P. AppA: a web server for analysis, comparison, and visualization of contact residues and interfacial waters of antibody-antigen structures and models. *Nucleic Acids Res.* **47**, W482–W489. 10.1093/nar/gkz358 (2019). 31069385 10.1093/nar/gkz358 PMC6602511

[40] Nguyen, M. et al. AllerCatPro 2.0: a web server for predicting protein allergenicity potential. *Nucleic Acids Res.* **50**, W36-43. 10.1093/nar/gkac446 (2022). 35640594 10.1093/nar/gkac446 PMC9252832

