# Supplementary material for: Lethal effects of ivermectin structures on malaria vectors and in silico analysis of interactions with their glutamate-gated chloride ion channels
Source: Sci Rep. 2026 Feb 10;16:8141. doi: 10.1038/s41598-026-39698-8 (PMC12960926; doi:10.1038/s41598-026-39698-8)
Supplement: Supplementary file 1 — Supplementary Information. [file 41598_2026_39698_MOESM1_ESM.docx]

**Supplementary Material**

**Table S1.** PCR primers used to amplify the coding sequences of *An. dirus* and *An. minimus* GluCl.

| **Species** | **Forward Primer** | **Reverse Primer** |
| --- | --- | --- |
| *An. dirus* | 5’- ATGGCCTCGGGCCATTTC -3’ | 5’ – TTAGTCTTCCTCCTCTTCGCG ‘3’ |
| *An. minimus* | 5’- ATGGCCTCGGGCCATTTC -3’ | 5’ – TTAGTCCTCCTCCTCTTCGCG ‘3’ |


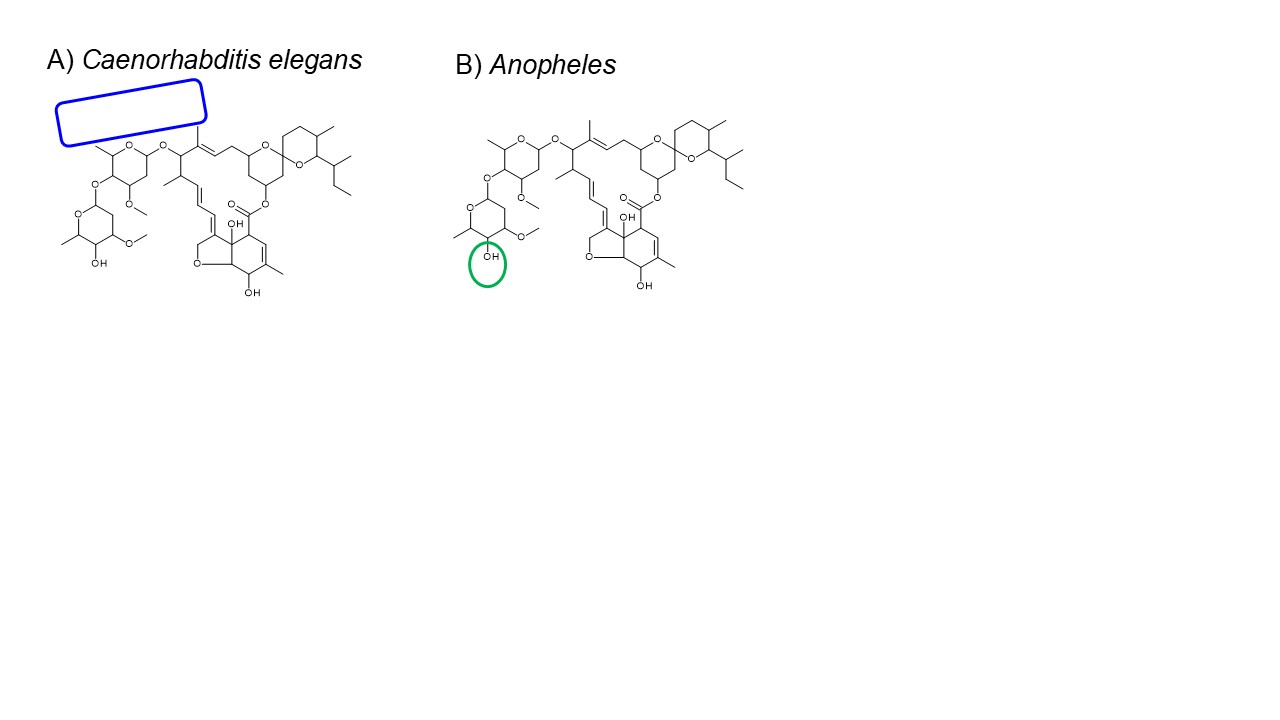


**Figure S1.** Ivermectin structure and primary site of interaction with the extracellular M2-M3 loop of *Caenorhabditis elegans* (A) as proposed previously^2^ and *Anopheles* (B) in the current docking simulations. The blue rectangle represents the first sugar ring and spiroketal region which form VDW interactions with the *C. elegans* M2-M3 loop^2^. The green circle highlights 4”-OH which forms the hydrogen bond with the *Anopheles* M2-M3 loop.


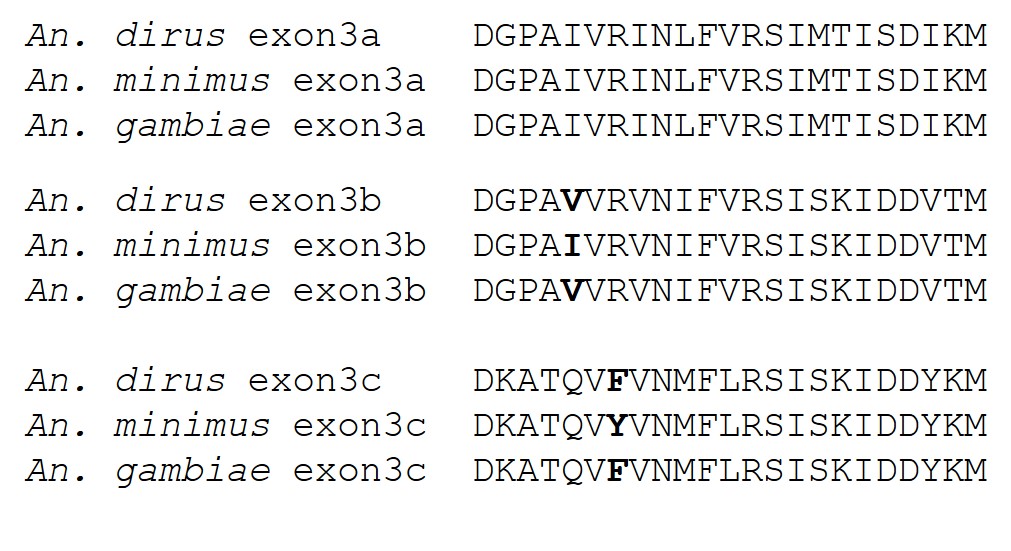


**Figure S2.** Alignment of alternatively spliced GluCl exon 3 sequences of *An. dirus*, *An. gambiae* and *An. minimus*. Residues that are not identical in all three species for each variant are shown in bold.


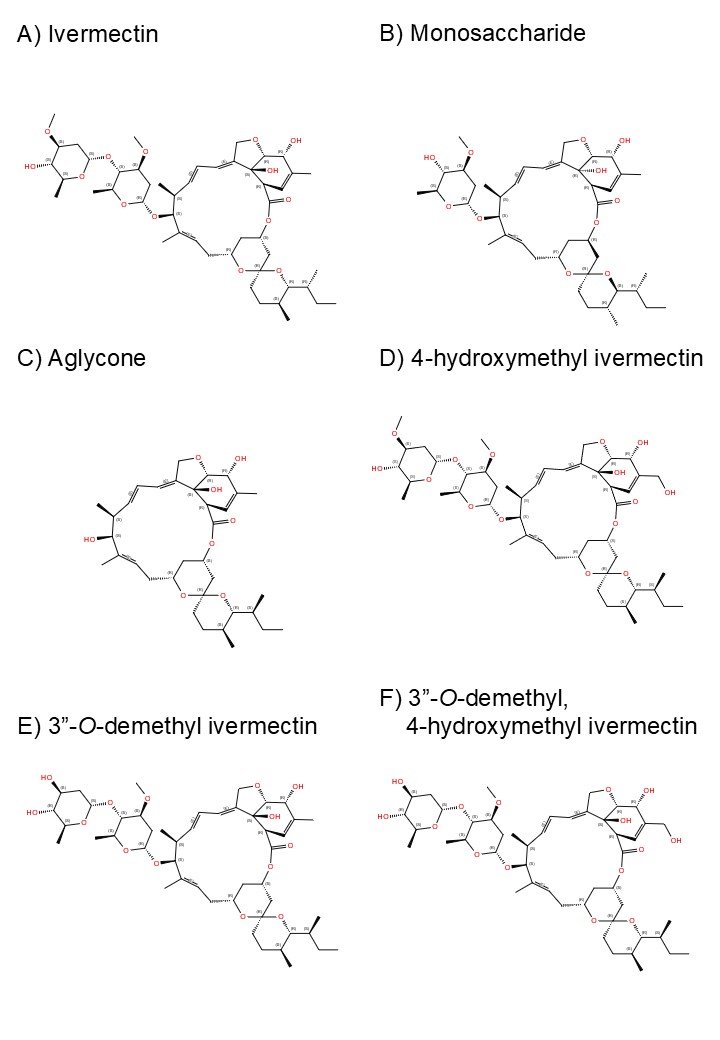


**Figure S3.** Stereochemical details for: ivermectin (A), monosaccharide (B), aglycone (C), 4-hydroxymethyl ivermectin (D), 3″-*O*-demethyl ivermectin (E), and 3″-*O*-demethyl, 4-hydroxymethyl ivermectin (F) are generated using Schrödinger Maestro, with chiral centers indicated by wedge and dashed bonds. Element colors follow the default Schrödinger convention.
